# Exploratory Effects of a Novel Nutraceutical on Senescence-Related Protein Biomarkers in Healthy Adults: A Pilot Proteomics Study

**DOI:** 10.3390/ijms27104406

**Published:** 2026-05-15

**Authors:** Sarah A. Blomquist, Gregory Kelly, Christopher R. D’Adamo, Chang Han, Haleigh Parker, Sara Adães, Colin R. Gardner, Abhimanyu Ardagh, Shawn Ramer, William Scuba

**Affiliations:** 1 Research and Development, Qualia Life Sciences, Carlsbad, CA 92008, USA; greg@qualialife.com (G.K.); sara@qualialife.com (S.A.); colin@qualialife.com (C.R.G.); abhi@qualialife.com (A.A.); shawn@qualialife.com (S.R.); 2 OvationLab, Richmond, VA 23220, USA; cdadamo@som.umaryland.edu; 3 Department of Family and Community Medicine, University of Maryland School of Medicine, Baltimore, MD 21201, USA; 4 LuminoDx Inc., San Diego, CA 92121, USA; chan@luminodx.com (C.H.); hparker@luminodx.com (H.P.)

**Keywords:** cellular senescence, SASP, senolytic, aging, longevity, healthspan, dietary supplement

## Abstract

Cellular senescence drives aging and age-related disease through the accumulation of senescent cells and their senescence-associated secretory phenotype (SASP). Emerging evidence suggests intermittent (“hit-and-run”) senolytic interventions may improve healthspan by reducing senescent cell accumulation and the SASP. Healthy adults aged 45–79 were recruited for a decentralized, single-arm pilot study (NCT06953518) evaluating 2 days of nutraceutical supplementation (Qualia Senolytic). Fingerstick blood samples and validated quality of life (QoL) questionnaire data were collected on days 0 and 7. Primary outcomes were SASP biomarkers measured by the Olink^®^ Target 48 Cytokine panel, including tumor necrosis factor (TNF), interleukin-1 beta (IL-1β), interleukin-8 (CXCL8), and vascular endothelial growth factor A (VEGFA). Protein data were analyzed using linear mixed models and Wilcoxon signed-rank tests. Seventy-one adults enrolled and 53 (74.6%) provided paired protein samples. No significant changes occurred in primary outcomes. Exploratory unadjusted analyses revealed significant reductions in the established senescence chemokines CXCL9 and CXCL10, as well as CCL8 and CXCL11, and increases in interleukin-17F and oncostatin M. QoL significantly improved without safety concerns, though results are expectation-sensitive. Preliminary findings support the feasibility of this decentralized approach and identify candidate SASP biomarker signals in healthy adults warranting validation in randomized, placebo-controlled trials.

## 1. Introduction

Cellular senescence is a stable form of cell cycle arrest that occurs in response to stressors like DNA damage, telomere shortening, oxidative stress, or other key hallmarks of aging [1]. While transient senescence can coordinate tissue growth and repair, promote immune cell homing, insulin secretion, and tumor suppression [2,3], accumulation of senescent cells can also contribute to aging and age-related disease. This leaves less functional stem and progenitor cells available for tissue repair and regeneration and inhibits healthy cell functions due to the detrimental microenvironment generated by the senescence-associated secretory phenotype (SASP) [1]. In fact, it is thought that the detrimental effects of senescent cells are largely driven by the SASP [1], which is composed of hundreds of protein and non-protein signaling molecules, including proteases, hemostatic factors, ceramides, bradykinins, extracellular matrix (ECM) components, and damage-associated molecular patterns (DAMPs) [2,4].

Cellular senescence and the SASP are increasingly recognized as central drivers to systemic aging and functional decline. Preclinical research shows that senescence is responsible for age-related decline in muscle stem cell regenerative function [5]. In fact, injection of senescent preadipocytes in young mice causes widespread physical dysfunction [6]. At the systemic level, 18% of plasma aging markers in humans overlap with SASP components [7], and these components increase with chronological age [8], acting as markers for age-related health risk [9].

The pivotal role of senescent cells in aging is further supported by interventional studies targeting both senescent cells and the SASP. The removal of senescent cells increases lifespan and healthspan in animal models [6,10,11,12,13,14,15]. Even without the removal of senescent cells, reduction in SASP signaling is also associated with modest improvements in lifespan [16] and notable improvements in healthspan [17,18,19].

The intention to develop senolytic drugs, or compounds that enable apoptosis of the otherwise resistant senescent cells, first appeared in a pilot study in 2004 [20]. Senescent cells use prosurvival pathways and other mechanisms to evade apoptosis. Thus, it was hypothesized they are more sensitive to inhibition of these prosurvival networks, which also led to the initial discovery and in vivo use of the pharmaceutical dasatinib and the natural compound quercetin as senolytics [21], as well as their eventual successful use in human clinical trials to clear senescent cells [22,23].

After senolytics disable senescent cell anti-apoptotic pathways (SCAPs) and induce senescent cell death, senescent cells can take weeks to re-accumulate in tissues, supporting the “hit-and-run” senolytic protocol originally described by Kirkland and collaborators at the Mayo Clinic [24]. This intermittent dosing of senolytics may also minimize off-target toxicity from cytotoxic agents. From another perspective of minimizing toxicity, natural senolytic phytochemicals may have the advantage of lower toxicity compared to synthetic compounds [25,26], as well as exhibiting multi-target senotherapeutic effects including the modulation of SASP pathways.

The flavonoids fisetin and luteolin, for example, and the alkaloid piperlongumine have also since been identified as natural senolytics [25]. Fisetin and luteolin also exhibit senomorphic activities by attenuating release of the SASP [27]. Overall, SASP modulation is gaining recognition as a complementary and alternative approach to senolytics [2], as senomorphic compounds can augment the SASP regulatory network and modify the tissue microenvironment to limit the spread of senescence to nearby healthy cells, a process known as paracrine senescence [28]. It is generally recommended that senomorphic drugs are administered on a continuous basis [29]; however, it has been proposed that intermittent dosing regimens may also be effective [30]. It has also been noted that continuous SASP repression can inhibit pathways necessary for the maintenance of tissue homeostasis and immune surveillance [31,32,33], highlighting the importance of balancing senotherapeutic efficacy with preservation of essential physiologic functions.

Due to the growing body of evidence that phytochemicals may support the clearance of senescent cells, SASP balance, and maintenance and rejuvenation of tissues, the overall objective of this hypothesis-generating pilot study was to examine changes in blood proteins related to cellular senescence, and changes in quality of life and aging symptoms, as a result of administration of a novel nutraceutical.

The nutraceutical evaluated in this study is a multi-ingredient dietary supplement formulated for short-duration, intermittent administration consistent with the hit-and-run concept. A two-day serving (12 capsules total) provides fisetin, Quercefit^®^ quercetin phytosome, Longvida^®^ curcumin extract, olive leaf extract, soybean seed isoflavones, luteolin, milk thistle silymarin, piperlongumine, and Senactiv^®^ (a combination of *Panax notoginseng* and *Rosa roxburghii* extracts). We hypothesized that two days of supplementation administered in a hit-and-run regimen would produce detectable changes from baseline in circulating SASP-associated proteins measured by the Olink Target 48 Cytokine panel, with pre-specified primary outcomes based on their consistent identification as senescence-associated biomarkers in the prior literature. Many SASP factors are secreted cytokines and chemokines that can enter systemic circulation; thus, measuring circulating protein levels via a fingerstick blood draw approach may provide a window into systemic senescence-associated inflammatory signaling. This pilot study was also conducted to address key uncertainties relevant to a future trial, including the sensitivity of Olink protein measurements following short-term supplementation, fingerstick blood draws, and identification of the most relevant proteins for prioritization in subsequent trials. Given the absence of prior clinical trials with this novel nutraceutical, this pilot study is essential to inform future investigations.

## 2. Results

### 2.1. Study Population and Feasibility Results

**Figure 1:**
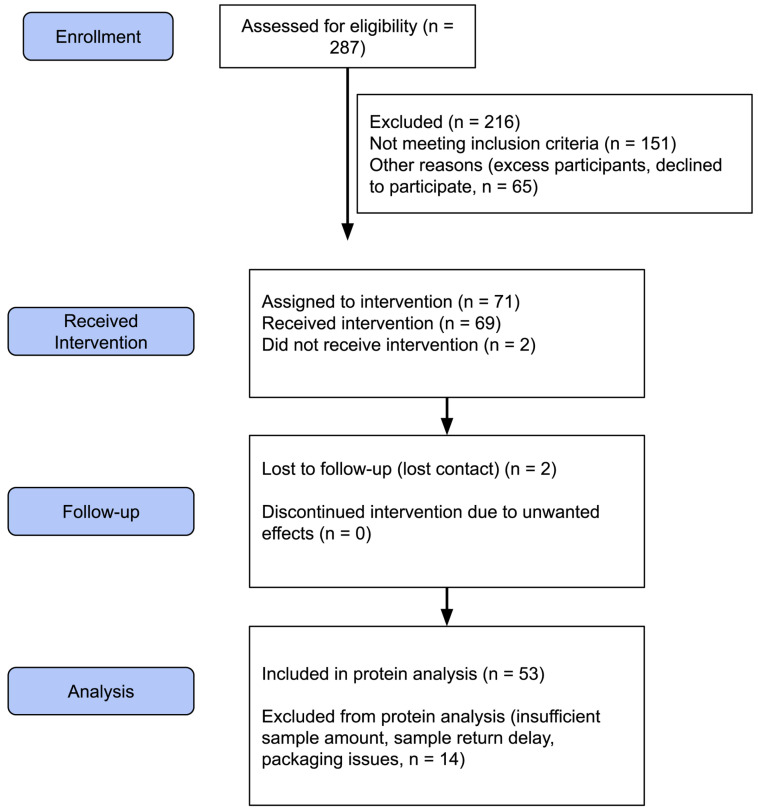
CONSORT flow diagram. Of 287 individuals assessed for eligibility, 80 consented and completed screening, 71 were enrolled, and 69 received the intervention. A total of 53 participants (74.6% of enrolled) were included in the primary proteomic analysis; 14 were excluded due to insufficient sample, sample return delay, or packaging issues. All 69 completers were included in secondary quality-of-life analyses.

Feasibility outcomes were evaluated post hoc across four domains: recruitment, retention, data completeness and intervention acceptability. Regarding recruitment, 287 individuals were assessed for eligibility and 216 were excluded (151 did not meet inclusion criteria; 65 were excess participants or declined to participate) (Figure 1). Of the 80 individuals who signed informed consent and completed screening, 71 were assigned to the intervention after completion of the initial fingerstick blood test (allocation rate: 88.8% of consented participants). Regarding retention, 69 of 71 assigned to intervention (97.2%) received the intervention and 2 were lost to follow-up (Figure 1). No individuals discontinued due to unwanted side effects, supporting the safety and tolerability of the intervention.

Of the 71 participants who were assigned to the intervention, 53 participants (74.6%) provided paired baseline and follow-up blood spot samples and were included in the primary protein analyses, while 14 were excluded due to insufficient sample amount, sample return delay, or packaging issues, identifying specimen logistics as a key challenge to address in a future full trial. For those included in the primary analysis, participants’ ages ranged from 45 to 79 (mean age 57.4 years), and were a mix of males (*n* = 24) and females (*n* = 29).

Regarding data completeness for secondary outcomes (*n* = 69), scale-level scoreability at baseline was 100% for AMFS, RAND-36, and safety and tolerability outcomes, and 98.6% for both DASS and FIFE. At the end of study (day 7), scoreability ranged from 81.2% (DASS) to 85.5% (AMFS, RAND-36), with 55 of 69 participants (79.7%) providing fully scoreable data at both timepoints. Within submitted surveys, item-level missingness was zero. Missing data pattern analysis indicated a Missing At Random (MAR) mechanism, as the 10 participants (14.5%) who completed no end of study surveys had significantly higher fatigue, poorer general health, and lower social functioning at baseline compared to completers (*p* = 0.005, 0.022, and 0.038, respectively). Regarding intervention acceptability, 68/69 participants who received the intervention submitted a rating to describe their satisfaction with the product and experience, with higher scores indicating higher acceptability. The mean rating was 4.7 out of 5.0.

### 2.2. Protein Analyses

**Figure 2:**
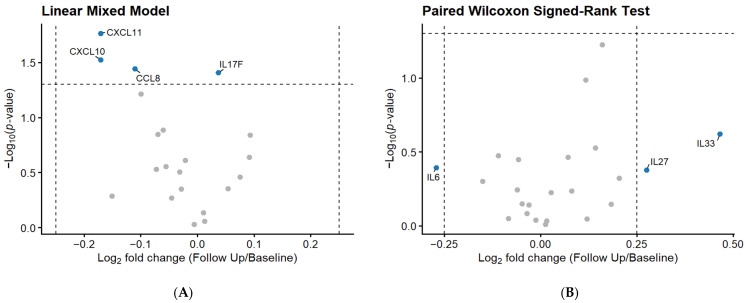
Volcano plot of protein changes from baseline to follow-up. (**A**) Results from linear mixed effects models (LMMs) fitted on log-transformed protein levels, adjusted for age, sex, time as a fixed effect, and participants as the random effect. (**B**) Results from paired Wilcoxon signed-rank analysis. Each panel includes only the subset of proteins deemed appropriate for the respective modeling approach based on diagnostic assessment. In both panels, dashed vertical and horizontal lines denote pre-specified thresholds of |log_2_FC| > 0.25 and *p* < 0.05, respectively. Unlabeled grey dots are additional proteins that did not meet *p*-value or |log_2_FC| thresholds.

The four pre-specified primary outcomes (TNF, IL1B, CXCL8, and VEGFA) were analyzed as confirmatory tests, while all remaining protein-level analyses (including non-primary cytokines, fold-change exploration, and sex-stratified subgroups) are exploratory and hypothesis-generating. Linear mixed model (LMM) and Wilcoxon analysis, after correction for multiple comparisons, demonstrated that compared to baseline (day 0), at follow-up (day 7) there were no significant changes in the primary outcomes (*p* > 0.38). Prior to false discovery rate (FDR) correction, unadjusted decreases in several senescence-associated secretory phenotype (SASP) biomarkers and secondary outcomes were noted, including CXCL11 (*p* = 0.017), CXCL10 (*p* = 0.029), and CCL8 (*p* = 0.036), as well as an increase in the immune marker IL-17F (*p* = 0.039) (Table 1, Figure 2). Among the proteins that did not satisfy LMM assumptions (*n* = 24) and were analyzed using paired Wilcoxon signed rank tests, none exhibited statistically significant differences. However, IL-6, IL-27 and IL-33 were notable in that they surpassed the log_2_ fold change threshold (>0.25), where decreases were observed in IL-6 (0.82 fold change, −17.1% decrease) and increases in IL-27 (1.21 fold change, 21.0% increase) and IL-33 (1.38 fold change, 38.1% increase) (Figure 2).

Exploratory Wilcoxon signed-rank analyses of proteins that met LMM assumptions identified an unadjusted significant increase in IL-17F and decrease in CXCL11, consistent with the LMM results (Table 1). In addition, consistent directional increases in the inflammatory biomarker OSM (*p* = 0.032) and decreases in the SASP biomarker CXCL9 (*p* = 0.032) were observed, with moderate rank-biserial effect sizes (r = 0.33 and −0.34, respectively), suggesting biologically meaningful group-level changes warranting confirmation. Results from FDR correction indicated all *p*-values were >0.38.

**Figure 3:**
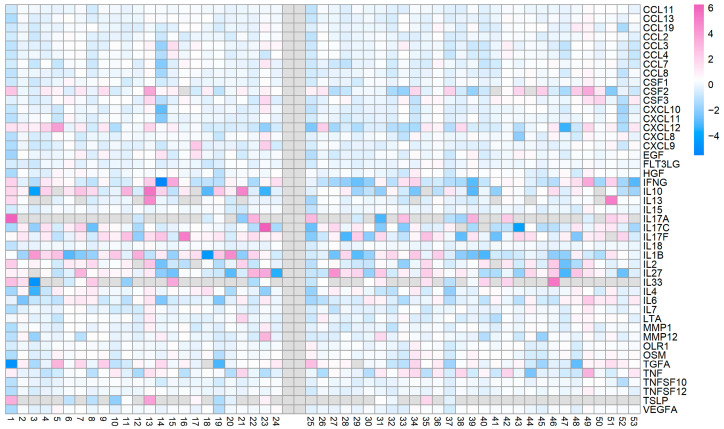
Log_2_ fold change in protein levels in individual participants. Heatmap illustrating protein responses from baseline to follow-up in individual participants, expressed as log_2_ fold change. Each column represents an individual participant and each row represents a protein assay, with participants grouped by sex: male (**left**), female (**right**). Color intensity reflects the magnitude and direction of change, with positive values indicating increases and negative values indicating decreases relative to baseline.

Interindividual variability in response to the intervention was observed both across the full population and within sex-specific subgroups, as evidenced by the heatmap in Figure 3 and summarized in Table S1.

### 2.3. Questionnaire Assessments

There were significant improvements at the end of the study compared to baseline (FDR *p*-value < 0.02) in the following: the 36-Item Short Form Health Survey (SF-36) categories vitality, emotional wellbeing, general health, and social functioning; the Depression Anxiety Stress Scale-21 (DASS-21) categories stress, depression, and overall; and the Aging Male/Female Symptoms Scale (AMFS) categories somatic, psychological, and sexual (Table S2). There were no significant changes in any of the safety and tolerability domains indicating a worsening of symptoms, suggesting the intervention was well tolerated. However, because this was a single-arm study without a placebo, these self-reported improvements are preliminary and expectancy-sensitive and cannot be interpreted as evidence of clinical efficacy.

## 3. Discussion

The results from this hypothesis-generating pilot study, leveraging a novel study design, indicate that acute supplementation with a commercially available nutraceutical intended to support senescent cell clearance and maintenance of healthy tissue yielded exploratory, unadjusted signals of reduction in the senescence-associated secretory phenotype (SASP) chemokines CCL8, CXCL9, CXCL10, and CXCL11. Prior human studies have established CXCL9 [34] and CXCL10 [34,35,36] as circulating protein biomarkers of cellular senescence. Although CCL8 and CXCL11 are not recognized as canonical senescence markers, they are known to be pathologically elevated as part of the pro-inflammatory profile that accompanies aging, age-related disease and the senescent phenotype [37,38,39,40,41]. The study also demonstrated strong feasibility across key domains, including high retention (97.2%), high intervention acceptability (mean rating 4.7/5.0), and high data completeness for patient-reported outcomes, supporting the practicality of this decentralized approach.

In addition, exploratory unadjusted significant increases were observed in the immune-associated markers IL-17F and oncostatin M (OSM). While both cytokines are often characterized as pro-inflammatory, transient increases may reflect immune activation associated with regenerative signaling, whereas sustained elevation is more commonly associated with pathological inflammation. To illustrate further, IL-17 signaling is known to increase with age, and experimental inhibition of this pathway in vivo reduces proinflammatory signaling and enhances skin health, without affecting markers of cellular senescence [42]. OSM, a member of the IL-6 protein family, also plays roles in inflammatory responses including wound healing, tissue regeneration and bone modeling; however, its prolonged expression may contribute to cellular senescence [43]. Notably, increases in some SASP factors after senolytic treatment have been shown to contribute to wound healing [44]. In this context, the observed elevations of IL-17F and OSM may indicate a beneficial immune or remodeling response, but more research is needed to understand the duration and biological significance of these protein changes in relation to cellular senescence.

Exploratory log_2_ fold change (FC) analysis also revealed candidate proteins with expression shifts (|log_2_FC| > 0.25), including decreases in IL-6 and increases in IL-27 and IL-33. IL-6 has been proposed as the most prominent pro-inflammatory cytokine of the SASP [45] and is an established biomarker of cellular senescence. IL-27 plays a role in immune regulation and inflammation and exhibits the ability to upregulate anti-inflammatory cytokines and suppress the pro-inflammatory tumor necrosis factor (TNF), another key SASP marker [46]. Dysregulation of IL-27 is linked to exacerbation of senescence [47]. IL-33 exhibits context-dependent effects, functioning either as a suppressor [48] or a promoter [49] of pathological senescence and inflammaging depending on tissue and disease state.

While no additional proteins demonstrated meaningful changes in the full sample, exploratory subgroup analysis suggests sex-differentiated responses. These sex-stratified analyses were hypothesis-generating observations rather than confirmatory tests. Females exhibited a greater number of proteins with unadjusted changes at follow-up, as well as a higher number showing trends (*p* < 0.10), as compared to males. Those trending included two senescence markers that were part of the primary outcome of the study: TNF and VEGFA. These observations were not corrected for multiple testing and should be regarded as candidate proteins and pathways that may be differentially responsive to the intervention and warrant further investigation in controlled studies.

The observed asymmetry between the null primary outcomes and the unadjusted directional signals among non-primary proteins likely reflects the evolving state of human SASP biomarker research. The four primary outcomes were selected based on their consistent identification as circulating senescence-associated biomarkers across prior proteomics, aging, and disease studies, as no single prior study has established a definitive primary SASP biomarker set for short-term senolytic interventions in healthy adults. The proteins showing unadjusted directional changes, such as the reductions in CXCL9, CXCL10, CXCL11 and CCL8, may represent more sensitive candidates for short-term senolytic intervention, while the primary outcomes may be less responsive to short-term hit-and-run regimens. CXCL9 and CXCL10 are also among the most reproducibly reported SASP chemokines in human proteomic studies of cellular senescence in aging and disease [34,35,36], and, together with CXCL11 and CCL8, share upstream regulation through the IFN-γ/JAK-STAT axis [50,51]. The present findings suggest the IFN-γ inducible chemokine axis may provide more sensitive candidate biomarkers for short-duration senolytic interventions in healthy populations.

In general, short- and long-term administration of senolytic and senomorphic therapies have been previously shown to modulate SASP biomarkers in both animal models [11,44,52,53,54] and human studies [23], although the magnitude and consistency of these effects varies widely by tissue, sex [53,55,56], disease context, and baseline senescence burden. In fact, most clinical studies have only reported directional changes in SASP factors. The one exception is the STAMINA pilot study, where six cycles of dasatinib and quercetin were administered to 12 older adults over 12 weeks. This study reported an average decrease in TNF-alpha of 3% (*p* = 0.04) when compared to baseline [57]. On the other hand, two human studies evaluating dasatinib and quercetin in idiopathic pulmonary fibrosis and in post-menopausal individuals have failed to show significant alteration of SASP markers at the population level [58,59]. However, exploratory analyses revealed that participants with a higher initial SASP burden or greater treatment-associated reduction in SASP markers exhibited more pronounced clinical and functional improvements, highlighting the heterogeneity and individual specificity in response to senolytic interventions. Consistent with this observation, it has been proposed that reductions in SASP biomarkers may serve as a key proxy for senolytic effectiveness and even clinical response [60].

Underlying the SASP is a complex crosstalk of multiple mechanisms, including the signaling pathways p53-p21 and p16-Rb, NF-κB, p38 MAPK, mTOR and cyclic GMP–AMP synthase–stimulator of interferon genes (cGAS–STING), as well as epigenetic changes and microRNA-mediated regulation [4]. Accordingly, therapeutic strategies have been developed to target these distinct components of senescent cell biology, including approaches that disrupt anti-apoptotic survival pathways (Bcl-2 family proteins, p53, and PI3k/Akt axis), interventions targeting lysosomal features (β-galactosidase activity) and immune modulation, and the use of senomorphics to suppress the SASP through pathways including NF-κB, p38 MAPK, and mTOR [27].

The novel nutraceutical used in this study combines senolytic and senomorphic ingredients and was formulated based on the growing body of evidence supporting the individual ingredients’ ability to modulate these underlying pathways and mechanisms. Fisetin, quercetin, and the structurally related flavonoid luteolin modify signaling pathways including PI3K/Akt, Bcl-2 family proteins, p53, p16, and mTOR, leading to senescent cell clearance, suppression of SASP signaling [61,62,63,64,65,66,67], and miRNA and epigenetic alterations [68,69]. The use of a combination of these structurally related flavonoids reflects preclinical evidence that these compounds engage distinct subsets of the senescent cell anti-apoptotic pathways (SCAPs) and SASP-regulatory networks; however, synergy for the combination in this study has not been formally demonstrated. Additional phytochemicals in the formula predominantly exhibit senomorphic activity, modulating inflammatory and stress-response pathways like NF-κB, p38 MAPK, cGAS–STING, JNK, and lysosomal β-galactosidase activity, with context-dependent senolytic effects in specific models [70,71,72,73,74,75,76,77,78,79,80,81,82]. Together, these complementary mechanisms may help to explain the observed effects on SASP-associated biomarkers in this study.

While this study also observed improvements in subjective health and aging symptoms, and a favorable safety profile of the supplement, these findings must be interpreted cautiously in light of several limitations. The absence of a placebo group in this study restricts more definitive conclusions. Furthermore, while the goal of this study was to evaluate the feasibility and preliminary signals of efficacy of the short-term “hit-and-run” style intervention, the short duration of the study limits the ability to assess long-term clinical relevance or impact on quality of life. Consistent with the hypothesis-generating intent of pilot research, the analytic sample was determined by feasibility considerations rather than by anticipated effect sizes and for hypothesis confirmation. The unadjusted directional signals identified should be regarded as candidate effects to be confirmed in adequately powered trials. This trial was also retrospectively registered on 23 April 2025 after the first participant had been enrolled on 18 April 2025; however, no changes were made to the study protocol. With regards to the technical limitations of the study, although Olink is well-established for relative protein quantification, the Target 48 calibration method is validated by the manufacturer in plasma and serum, so the dried blood spot (DBS)-derived absolute concentrations should be regarded as semi-quantitative. Importantly, this semi-quantitative outcome only limits interpretation of the absolute protein values and does not affect the paired within-participant change estimates reported.

Additionally, senescent cell burden was not directly measured and changes in SASP biomarkers cannot be solely attributed to senescent cell clearance or suppression. However, many of the SASP biomarkers under study have been associated with senescent cell clearance or suppression. We also acknowledge that the single post-treatment timepoint falls at the early edge of the documented window (3–10 days) [83] for SASP changes and may underestimate the peak response that a later or repeated assessment would capture. Time-varying lifestyle factors over the 7-day window (e.g., changes in diet, exercise, sleep, stress, or acute illness) were also not formally measured and could contribute to residual variability. Future controlled trials should include later timepoint assessments, as well as structured lifestyle monitoring, to more accurately capture the dynamic range of post-senolytic SASP modulation. With regards to trial feasibility, 16 of 69 dosed participants did not contribute paired protein samples—14 due to specimen logistics (sample volume, return delay, or packaging), and 2 were lost to follow-up. Because the missingness mechanism for this dataset was not characterized, selection bias cannot be excluded. The specimen-related failure logistics observed, however, elucidate concrete improvements for future decentralized dried blood spot (DBS)-based studies. These include providing multiple DBS cards per participant for redundancy, pairing existing visual fill-line and pre-assembled kit materials with detailed video instruction, implementing automated reminders with a defined return window, and offering real-time troubleshooting support for first-time users. Lastly, interindividual variability in baseline senescent cell burden, phytonutrient metabolism [84], and biological response to senolytic interventions may contribute to differential benefit across individuals. These potential sources of heterogeneity were not measured in the present study and therefore could not be determined as modifiers of response or used to determine which individuals may most likely benefit from the intervention. While this study did identify potential sex-related differences, other characteristics should be evaluated in future studies.

In conclusion, this hypothesis-generating pilot study suggests that acute supplementation with the novel nutraceutical was associated with exploratory unadjusted reductions in multiple SASP biomarkers, including established indicators of cellular senescence like CXCL9 and CXCL10. These findings represent exploratory signals that did not survive false discovery rate correction and require confirmation in controlled trials. Additional exploratory fold change analyses revealed shifts in IL-6 and other proteins that may warrant further exploration. Hypothesis-generating subgroup analyses also revealed candidate sex-differentiated effects, highlighting a heterogeneity of responses not captured at the population level. Although subjective improvements in quality of life were noted, these findings should be interpreted cautiously and not taken as clinical efficacy due to expectation-sensitivity, the absence of a placebo control, and short study duration. Taken together, these preliminary findings support the need for future controlled studies to better understand mechanisms, evaluate long-term clinical relevance, and identify responder phenotypes most likely to benefit from the intermittently administered senolytic and senomorphic nutraceutical intervention.

## 4. Materials and Methods

### 4.1. Study Design and Intervention

**Figure 4:**
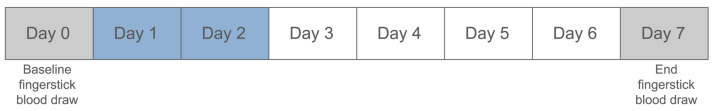
Experimental design diagram. Blue filled boxes represent days that the novel nutraceutical was taken. Gray filled boxes represent days that participants performed the fingerstick blood draw and completed the 36-Item Short Form Health Survey (SF-36), Frailty Index for Elders (FIFE), Depression Anxiety Stress Scale-21 (DASS-21), Aging Male/Female Symptoms Scale (AMFS), and the custom safety and tolerability symptoms questionnaire.

This decentralized, single-arm, open-label pilot trial was conducted between April 2025 and May 2025. The trial was registered at ClinicalTrials.gov (Identifier: NCT06953518) on 23 April 2025, after the first participant was enrolled on 18 April 2025, due to administrative processing delays. In alignment with the “hit-and-run” senolytic approach, all participants were assigned to receive a 2-day supply (12 capsules total) of Qualia Senolytic, reflecting the product’s intended short-duration dosing protocol. These capsules were to be taken at any time in the morning with or without food, during days 1 and 2 of the study. The participants were instructed to complete an at-home fingerstick blood collection to measure protein levels at day 0 and at day 7 (Figure 4). Both the study product and fingerstick blood collection kits were drop-shipped to enrolled participants. The primary objective was to investigate the impact of the novel nutraceutical on changes in blood of four commonly measured proteins consistently associated with SASP, tumor necrosis factor (TNF), interleukin-1β (IL1B), interleukin-8 (CXCL8), and vascular endothelial growth factor A (VEGFA). Secondary outcomes were changes in levels of the remaining 41 proteins measured from the protein panel and findings from validated quality-of-life-related questionnaires.

### 4.2. Participants

For this decentralized clinical trial, participants were recruited using an online recruitment platform. Individuals expressing interest in the study were asked to complete a prescreen survey to determine their eligibility, and adults who met the inclusion criteria were informed of the intervention details and asked to sign informed consent. Healthy subjects aged 45–79 were included in the study if they were willing to: provide a valid cell phone number and receive text communications, complete questionnaires associated with the study, not start any new supplements during the study period, continue taking any supplements they regularly use during the study period, and self-administer the Olink fingerstick test at baseline and follow-up. Exclusion criteria included: women who are pregnant, breastfeeding, or planning to become pregnant during the trial, those with a known food intolerance/allergy to any ingredients in the product, those with psychiatric conditions (including those taking MAOIs, SSRIs, or other medications), neurologic disorders, endocrine disorders, cancer, having had a cardiovascular event in the past 6 months, or currently taking immunosuppressive therapy, and individuals who are unable or unwilling to comply with study procedures or lacking the ability to consent.

### 4.3. Ethics

This study protocol was approved by the Advarra Institutional Review Board (protocol number: Pro00084871) and adheres to international ethical standards for human research, including the principles outlined in the 2024 Declaration of Helsinki [85]. Informed consent for participation was obtained from all subjects involved in the study. All authors had full access to the study data and reviewed and approved the final manuscript.

### 4.4. Study Product

Qualia Senolytic (Qualia Life Sciences LLC, Carlsbad, CA, USA) is a dietary supplement formulated to support the elimination of senescent cells, tissue rejuvenation and maintenance, and healthy aging. All ingredients were selected due to known senolytic activity or ability to modulate the senescence-associated secretory phenotype (SASP). The serving size of 6 dietary capsules is recommended to be taken twice, over two consecutive days, once a month. Six capsules provide the following: 1400 mg fisetin (from Rhus succedanea stem extract), 750 mg Quercefit^®^ quercetin phytosome (from *Sophora japonica* L. flower extract/phospholipid complex from sunflower), 400 mg Longvida^®^ (curcumin extract from *Curcuma longa* root, standardized to 23% curcuminoids, Verdure Sciences Inc., Noblesville, IN, USA), 250 mg olive leaf extract standardized to 40% oleuropein, 200 mg soybean seed extract standardized to 40% isoflavones, 150 mg luteolin (from *Sophora japonica* L. flower bud extract), 125 mg milk thistle fruit extract standardized to 58% silymarin by HPLC (80% by spectrophotometry), 50 mg piperlongumine (from *Piper longum* root extract), and 50 mg Senactiv^®^ (from *Panax notoginseng* root extract and *Rosa roxburghii* fruit extract, NuLiv Science USA Inc., Brea, CA, USA), providing 5 mg ginsenoside Rg1. All of the ingredients meet the criteria for dietary supplements established by the FDA, and all dosages are safe.

### 4.5. Protein Level Testing

The abundance of proteins was measured using the Olink^®^ Proximity Extension Assay (PEA) platform (Olink Proteomics AB, Uppsala, Sweden), as described previously [86]. In this assay, pairs of antibodies labeled with complementary DNA oligonucleotides bind to target proteins. Upon target recognition by pairs, the conjugated oligonucleotides are extended by a polymerase. The amount of extended oligonucleotides for each antibody pair is used to measure the amount of detected proteins and is recorded by real-time PCR. The PEA platform offers high sensitivity (femtomolar range) and has been validated for use with dried blood spot eluates, supporting its suitability for decentralized, home-based sample collection in this study [86].

Olink offers pre-specified protein panels within its Target 48 series, focusing on analysis of immune and inflammation-related proteins. This pilot study utilized the Olink Target 48 Cytokine panel that covers 45 proteins, including interleukins, interferons, chemokines, tumor necrosis factors, and growth factors. The Target 48 panel was selected based on the recent proteomics literature identifying circulating senescence-associated protein biomarkers and their associations with disease, aging, and risk of death, as it included the greatest number of relevant proteins (e.g., CCL2, CCL3, CCL5, CXCL8, CXCL9, CXCL10, IL1B, IL6, IL-17A, HGF, VEGFA, TNF) [34,35,36,52,87,88]. The proteins most consistently reported across studies, including VEGFA, IL1B, TNF and CXCL8, were designated as primary outcomes for the study. Although there was little human research to guide the timing of our study, we primarily based on a human dasatinib and quercetin study that employed multiple 2-day dosing regimens over time and noted changes in circulating plasma proteins, including IL-6 and VEGF, 3–10 days after the final study drug dose [83]. Endogenous cytokines as a class are also characterized by rapid plasma clearance, with half-lives on the order of minutes to hours [89]. Because the post-intervention sample was collected 5 days after the final dose, this window is hypothesized to be sufficient for turnover of pre-existing circulating cytokines, such that differences from baseline represent altered ongoing production rather than acute supplement–cytokine effects.

### 4.6. Dried Blood Spot Collection, Preparation, and Olink Processing

Participants prepared dried blood spot (DBS) samples by washing and drying their hands, punching a fingertip with a sterile lancet, discarding the first drop of blood, and applying subsequent drops to pre-labeled filter paper circles until saturated. Cards were air-dried at room temperature for at least three hours, then packed with desiccant in biohazard bags and returned to the laboratory for analysis. Upon arrival at the laboratory, samples were stored in a −80 °C freezer until they could be processed as a batch. For DBS sample preparation, 3 mm punches were collected from blood spots. Each punch was transferred to a LoBind microcentrifuge tube containing 20 µL of elution buffer (1X PBS with 0.05% TWEEN 20 and 1X protease inhibitors). To limit carry-over contamination between samples, punching from an empty area of the sampling card was performed between every sample. Samples were incubated on a tube shaker for 1 h at room temperature at 600 rpm to ensure complete elution. The eluates were immediately used for Olink analysis. Olink NPX Signature software (version 1.17.0; Olink Proteomics AB, Uppsala, Sweden) was used for quality control and data analysis. Normalized protein concentrations were reported as Normalized Protein eXpression (NPX) units on a log_2_ scale, enabling comparison of relative protein abundance across study groups. The NPX values were also converted to absolute concentrations (pg/mL) based on an internal standard curve.

### 4.7. Check-Ins and Safety and Tolerability

Participants completed check-in questions in a self-reported diary to assess adherence and side effects. They were asked to record how many capsules they took, the time of day they were taken, and whether the capsules were taken with food. Participants were also asked to complete a safety and tolerability survey at baseline (day 0) and follow-up (day 7) that assessed common symptoms associated with interventions and nocebo effects [90], including changes in anxiety and mood, headache, stomach and GI, dizziness, itching, and sexual health.

### 4.8. Survey Assessments

At baseline (day 0) and at the end of the study (day 7), participants were asked to complete the following validated questionnaires to assess secondary exploratory outcomes related to symptoms of general health, quality of life, frailty, depression and anxiety, and aging: Research and Development Corporation (RAND) 36-Item Short Form Health Survey (SF-36) [91], Frailty Index for Elders (FIFE) [92], and the Depression Anxiety Stress Scale-21 (DASS-21) [93]. All participants were also asked to complete a modified Aging Male/Female Symptoms Scale (AMFS). The AMFS is a gender-neutral 15-item adaptation of a validated aging symptoms scale for men, the Aging Male Symptoms Scale (AMS) [94]. In this survey, male-specific items (questions 15–16) are removed and question 14 is modified to be gender-neutral. Subscale scores (somatic, psychological, and sexual) were computed using parent AMS assignments, with male-specific items 15–16 omitted from the sexual subscale. In two previous studies, we employed the gender-neutral AMFS for female participants [95,96]. In this study, this single-instrument approach was chosen to enable direct, sex-comparable scoring on a common scale rather than relying on parallel but non-identical scales.

The RAND SF-36 consists of 36 questions covering eight health domains, with items scored on Likert-type scales that are transformed to domain scores ranging from 0 to 100, with higher scores indicating a more favorable state. The FIFE employed in this study is 10 questions and uses a cumulative deficit model typical of frailty indices, where each of the 10 items is scored as present (1) or absent (0), yielding a total score ranging from 0 to 10, and higher values indicating greater frailty. The DASS-21 contains 21 questions with three subscales covering depression, anxiety, and stress, each category with 7 questions. Responses are on a 4-point Likert scale ranging from 0 to 3, where higher total scores indicate worse symptoms. Lastly, the AMFS is scored by rating answers to questions from 1 to 5, and then summing all item scores to obtain a total symptom severity score, with higher scores indicating worse symptoms.

### 4.9. Statistical Analysis

Descriptive statistics were computed to characterize the study sample. The normality of study outcomes was assessed using visual methods, such as histograms and Q-Q plots, alongside the Shapiro–Wilk test. To analyze changes in protein levels comparing baseline (day 0) to follow-up (day 7), linear mixed-effects models (LMMs) were used with age and sex as covariates, time (baseline and follow-up) as a fixed effect, and participant as the random effect to account for repeated measures. Protein level data were log-transformed prior to fitting the LMM. For each protein, LMM assumptions (including normality and homoscedasticity of residuals) were evaluated using residual histograms, Q-Q plots, and plots of residuals versus fitted values. For proteins that did not meet LMM assumptions despite log transformation, paired Wilcoxon signed-rank tests were performed on raw data. Primary model choice for each protein was guided by standard diagnostic checks. In addition, paired Wilcoxon signed-rank tests were conducted as complementary, exploratory analyses for proteins that met LMM assumptions, with rank-biserial effect sizes used to assess the strength and consistency of paired changes. Exploratory sex-differentiated subgroup analyses were also included, as quercetin has previously been shown to elicit sex-dependent differential effects on vascular senescence [55]. All estimates are reported as percent change with 95% confidence intervals, with LMM-based estimates obtained by back-transformation of log-transformed models and Wilcoxon-based estimates calculated from median within-participant percent changes. To account for multiple testing, *p*-values were adjusted using the Benjamini–Hochberg false discovery rate (FDR) procedure separately within each method (LMM or Wilcoxon) across all 45 proteins. Sex-stratified subgroup analyses for protein and survey data were also FDR-adjusted within each sex separately and reported as exploratory signals. The same Benjamini–Hochberg FDR Threshold (*q*-value = 0.05) was applied within each method (LMM and Wilcoxon). In line with guidance for pilot studies, no a priori power analysis was performed, as the primary intent was to assess feasibility and data quality rather than estimate effect sizes. Sample size was based on logistical considerations.

Volcano plots were used to visualize results from both model approaches by plotting log_2_ fold change (log_2_FC) against −log_10_(*p*-value). For the LMM, log_2_FC was derived by rescaling the model-estimated time effect from the natural log scale. For the Wilcoxon method, log_2_FC was calculated as the median of per-participant log_2_(Follow-up/Baseline) values. Exploratory thresholds of |log_2_FC| > 0.25 (representing ~1.19 fold change) or *p* < 0.05 were applied across plots to highlight effects of interest. Additionally, within-participant log_2_FC values for each protein were visualized using a heatmap across all protein assays, stratified by sex, to illustrate the distribution and direction of individual-level changes.

Changes in questionnaire outcomes from baseline to follow-up were evaluated using paired Wilcoxon signed-rank tests, which were selected due to the non-normally distributed nature of the data. Baseline and follow-up intervention values were summarized using medians, effect estimate was reported as the median of within-participant differences, and standardized effect sizes were quantified using the rank-biserial correlation (r). Wilcoxon test statistics were expressed using the normal approximation (z statistic). To examine sex-specific patterns on a single common instrument, AMFS total and subscale scores (somatic, psychological, and sexual) were analyzed within male and female subgroups using the same Wilcoxon procedure.

All statistical analyses were performed in RStudio v2026.01.0 (Posit, Boston, MA, USA) with R version 4.5.0 (11 April 2025 ucrt). Generative artificial intelligence (ChatGPT) was used to help generate and edit code for data analysis. The deidentified, raw study datasets analyzed for this study can be found in Zenodo (https://doi.org/10.5281/zenodo.19372850, accessed on 6 April 2026).

### 4.10. Feasibility Outcomes

Four domains were assessed for feasibility, including participant recruitment, retention, data completeness, and intervention acceptability. Recruitment and retention rates were calculated as proportions of screened, consented, and assigned participants who received the intervention and completed follow-up. Data completeness was evaluated at the scale level as the proportion of enrolled participants with fully scorable instruments at each timepoint. Missing data patterns were assessed by comparing baseline characteristics between completers and non-completers. Acceptability was captured using a post-intervention participant satisfaction rating.

## Figures and Tables

**Table 1:** Summary statistics highlighting unadjusted significant protein changes from baseline to follow-up. This table reports proteins showing statistically significant changes (*p* < 0.05) based on either linear mixed models (LMMs) or paired Wilcoxon signed-rank analysis. For the LMM, percent changes from baseline and corresponding 95% confidence intervals (as % change) were derived by back-transforming model estimates from the log scale. For Wilcoxon analyses, percent changes were calculated from the median within-participant percent changes. All FDR-adjusted *p*-values were *p* > 0.38. Note the Wilcoxon results represent complementary, exploratory re-analyses of proteins that met LMM assumptions and should not be interpreted as an independent panel of tests. Where the same protein appears in both sections, this reflects agreement between two analyses of the same data.

Model	Protein	*p*-Value †	Percent Change from Baseline	95% Confidence Interval
LMM (Primary)	CXCL11	0.017	−11.18	(−19.15, −2.42)
	CXCL10	0.029	−11.16	(−19.91, −1.47)
	CCL8	0.036	−7.37	(−13.59, −0.70)
	IL-17F	0.039	2.5	(0.19, 5.00)
Wilcoxon (Complementary)	OSM	0.032	14.46	(7.09, 28.22)
	CXCL9	0.032	−9.18	(−17.04, −0.25)
	IL-17F	0.037	72.19	(−0.66, 103.38)
	CXCL11	0.047	−4.86	(−11.90, −0.30)

† FDR-adjusted *p* > 0.38 for all proteins.

## Data Availability

The deidentified, raw study datasets analyzed for this study can be found in Zenodo (https://doi.org/10.5281/zenodo.19372850, accessed on 6 April 2026).

## Supplementary Materials

The following supporting information can be downloaded at: https://www.mdpi.com/article/10.3390/ijms27104406/s1.

## Abbreviations

The following abbreviations are used in this manuscript:

AFS	Aging Female Symptoms Scale
AMFS	Aging Male/Female Symptoms Scale (combined reference to AMS and AFS)
AMS	Aging Male Symptoms Scale
cGAS–STING	Cyclic GMP–AMP synthase–stimulator of interferon genes
CCL2	C-C motif chemokine ligand 2
CCL3	C-C motif chemokine ligand 3
CCL5	C-C motif chemokine ligand 5
CCL8	C-C motif chemokine ligand 8
CONSORT	Consolidated Standards of Reporting Trials
CXCL8	C-X-C motif chemokine ligand 8 (interleukin-8)
CXCL9	C-X-C motif chemokine ligand 9
CXCL10	C-X-C motif chemokine ligand 10
CXCL11	C-X-C motif chemokine ligand 11
DAMPs	Damage-associated molecular patterns
DASS-21	Depression Anxiety Stress Scale-21
DBS	Dried blood spot
DNA	Deoxyribonucleic acid
ECM	Extracellular matrix
FC	Fold change
FDA	U.S. Food and Drug Administration
FDR	False discovery rate
FIFE	Frailty Index for Elders
GI	Gastrointestinal
HGF	Hepatocyte growth factor
HPLC	High-performance liquid chromatography
IL-1β	Interleukin-1 beta
IL-6	Interleukin-6
IL-17F	Interleukin-17F
IL-27	Interleukin-27
IL-33	Interleukin-33
IL-17A	Interleukin-17A
IFN-γ	Interferon gamma
JAK-STAT	Janus Kinase-Signal Transducer and Activator of Transcription
JNK	c-Jun N-terminal kinase
LMM	Linear mixed model
log_2_FC	Log base-2 fold change
MAOIs	Monoamine oxidase inhibitors
MAPK	Mitogen-activated protein kinase
MAR	Missing at random
MCI	Mild cognitive impairment
miRNA	MicroRNA
mTOR	Mechanistic target of rapamycin
NF-κB	Nuclear factor kappa-light-chain-enhancer of activated B cells
NLRP3	NLR family pyrin domain containing 3
NPX	Normalized protein expression
OSM	Oncostatin M
PBS	Phosphate-buffered saline
PCR	Polymerase chain reaction
PEA	Proximity extension assay
PI3K	Phosphoinositide 3-kinase
QoL	Quality of life
RAND SF-36	RAND 36-Item Short Form Health Survey
RNA	Ribonucleic acid
ROS	Reactive oxygen species
SASP	Senescence-associated secretory phenotype
SCAPs	Senescent cell anti-apoptotic pathways
SF-36	36-Item Short Form Health Survey
SIRT1	Sirtuin 1
SSRIs	Selective serotonin reuptake inhibitors
TNF	Tumor necrosis factor
TXNIP	Thioredoxin-interacting protein
VEGF	Vascular endothelial growth factor
VEGFA	Vascular endothelial growth factor A
