# Supplementary material for: Exploratory Effects of a Novel Nutraceutical on Senescence-Related Protein Biomarkers in Healthy Adults: A Pilot Proteomics Study
Source: Int J Mol Sci. 2026 May 15;27(10):4406. doi: 10.3390/ijms27104406 (PMC13207486; doi:10.3390/ijms27104406)
Supplement: Supplementary file 1 [file ijms-27-04406-s001.zip › ijms-4265435-supplementary.pdf]

Supplementary Table S1: Sex-Stratified Effects Highlighting Unadjusted Protein Changes from Baseline to Follow Up. Changes in protein concentrations from baseline to follow up assessed using linear mixed effects (LMM) models and Wilcoxon signed-rank tests. Estimates are summarized as percent change from baseline with 95% confidence intervals (as % change), together with exploratory unadjusted p-values < 0.10 from each analysis method. All FDR-adjusted p-values were > 0.20.

Model	Sex	Protein	P-value†	Percent Change from Baseline	95% Confidence Interval
LMM	Male	IL17F	0.004	4.64	(1.72, 7.65)
		FLT3LG	0.074	-8.50	(-16.59, 0.37)
		IL2	0.080	0.75	(-0.05, 1.5)
	Female	OSM	0.027	14.57	(2.39, 28.20)
		IL17C	0.033	-14.44	(-25.08, -2.29)
		CXCL10	0.059	-11.73	(-21.93, -0.19)
		CXCL11	0.071	-11.02	(-21.15, 0.39)
		OLR1	0.084	10.02	(-0.79, 22.02)
		IL10	0.086	-4.29	(-8.76, 0.39)
		TNF	0.086	15.91	(-1.32, 36.17)
		HGF	0.095	11.34	(-1.30, 25.62)
Wilcoxon	Male	IL17F	0.007	80.50	(44.48, 126.37)
		CXCL9	0.051	-14.79	(-23.74, 1.29)

		CCL19	0.064	-7.41	(-14.48, 2.16)
	Female	OSM	0.040	25.56	(7.74, 40.30)
		CXCL10	0.051	-11.65	(-19.24, -2.72)
		IL17C	0.070	-26.29	(-60.88, -5.49)
		HGF	0.079	17.37	(9.67, 21.32)
		OLR1	0.085	11.37	(3.73, 19.34)
		VEGFA	0.097	13.57	(-2.55, 19.49)

† FDR-adjusted $p > 0.20$ for all proteins.

Supplementary Table S2: Summary of Significant Findings in RAND SF-36, DASS-21 and AMFS Outcomes from Baseline to Follow Up. This table presents paired Wilcoxon signed-rank analysis results demonstrating significant improvements from baseline to follow up ($p < 0.05$). Significant findings were only observed in the 36-Item Short Form Health Survey (SF-36), the Depression Anxiety Stress Scale-21 (DASS-21), and the Aging Male/Female Symptoms Scale (AMFS) for the male subgroup only. For each survey outcome, the table reports the median baseline and follow up values, median difference, Wilcoxon test statistics using the normal approximation (z stat), and standardized effect sizes as rank biserial correlation (r).

Survey	Category (n)	Median Baseline	Median Follow Up	Median Difference	Z stat	Rank biserial correlation (r)	p-value
**RAND SF-36**	Vitality (59)	55.0	65.0	5	4.39	0.64	<0.001
	Emotional Wellbeing (59)	76.0	84.0	4	2.99	0.33	0.002
	General health (59)	80.0	85.0	0	2.86	0.33	0.004
	Social functioning (59)	87.5	100	0	2.65	0.51	0.009
**DASS**	Stress (55)	4.0	2.0	-1	-3.88	-0.45	<0.001
	Depression (55)	2.0	0	0	-2.49	-0.52	0.014
	Overall (55)	5.0	3.0	-2	-3.18	-0.48	0.001
**AMFS,**	Somatic	15.5	12.0	-3	-3.85	-0.75	0.001

**Male Subgroup**	(26)						
	Psychological (26)	8.0	8.0	-1	-2.71	-0.68	0.007
	Sexual (26)	4.0	3.5	-1	-3.06	-0.64	0.002
